# On-site and visual detection of genotype VII Newcastle disease virus based on RT-RPA and CRISPR/Cas13a

**DOI:** 10.3389/fvets.2026.1870607

**Published:** 2026-09-03

**Authors:** Yaping Huang, Wei Li, Yuhui Song, Qizhang Liang, Qiuling Fu, Ningning Cheng, Yong Xie, Guanghua Fu

**Affiliations:** 1 Minjiang Teachers College, Fuzhou, China; 2 Institute of Animal Husbandry and Veterinary Medicine/Fujian Key Laboratory for Control and Prevention of Avian Diseases, Fujian Academy of Agricultural Sciences, Fuzhou, China; 3 College of Bee Science and Biomedicine, Fujian Agriculture and Forestry University, Fuzhou, China; 4 College of Animal Sciences, Fujian Agriculture and Forestry University, Fuzhou, China

**Keywords:** genotype VII, lateral flow dipstick, Newcastle disease virus, RT-RPA-CRISPR/Cas13a, virulent

## Abstract

**Introduction:**

Newcastle Disease Virus (NDV) is not only a significant and persistent threat to the healthy development of poultry industry, but also an important pathogen endangering food safety and harboring potential zoonotic risks.

**Methods:**

In this study, a rapid and highly sensitive diagnostic assay was established for genotype VII NDV by integrating reverse transcription recombinase polymerase amplification (RT-RPA) with the clustered regularly interspaced short palindromic repeats (CRISPR)/Cas13a system, in combination with a lateral flow dipstick (LFD) for visual signal readout. Specific RT-RPA primers were designed based on the fusion (F) gene of genotype VII NDV, and CRISPR RNAs (crRNAs) were constructed to establish a simplified CRISPR/Cas13a-based diagnostic workflow targeting the fusion (F) gene of genotype VII NDV.

**Results:**

The optimized detection platform integrates RT-RPA amplification at 39 °C for 20 min and CRISPR/Cas13a-mediated cleavage at 37 °C for 20 min. The final results can be quantified by fluorescence signals or visualized via LFD band within 5 min, with the total detection procedure completed in less than 45 min. Performance evaluation demonstrated that this assay achieved a limit of detection (LOD) as low as 6 copies per microliter of input and high specificity, with no cross-reactivity against other common avian pathogens. Clinical validation of 154 samples showed 100% concordance between the RT-RPA-CRISPR/Cas13a-(LFD) assay and RT-PCR sequencing for genotype VII NDV detection.

**Discussion:**

This method requires no sophisticated instruments and enables simple operation. It provides a field-deployable tool for rapid on-site detection of genotype VII NDV, which is suitable for preliminary screening in primary laboratories, and holds promising application potential for the early surveillance and food safety monitoring of virulent NDV strains.

## 1 Introduction

Newcastle Disease (ND) is a highly contagious viral infection that can lead to high morbidity and mortality in both domestic poultry and wild bird populations (1–4). The causative agent, Newcastle disease virus (NDV), also known as avian paramyxovirus type 1 (APMV-1), belongs to the family *Paramyxoviridae*, subfamily *Avulavirinae*, and genus *Orthoavulavirus*, and possesses a single-stranded, negative-sense RNA genome of approximately 15.2 kb (5–7). Based on clinical signs and lesions in infected chickens, NDV isolates are divided into three pathotypes: velogenic (highly virulent), mesogenic (moderately virulent), and lentogenic (avirulent) (1). Although NDV constitutes a single serotype, it displays extensive genetic diversity and is classified into two classes (Class I and II). Class I consists mainly of avirulent strains from wild birds, whereas Class II comprising both avirulent and virulent isolates and is further subdivided into 21 genotypes (I-XXI) (8, 9). Since 1985, genotype VII isolates of Class II NDV have driven panzootics (10) and appear to constitute the predominant causative agents of contemporary ND outbreaks throughout Eurasia, Africa, and South America, posing the most significant threat to poultry production, even though genotype VI strains are globally distributed (11–13).

Molecular and innovative technological approaches, such as RT-PCR, RT-qPCR, RT-LAMP, have been employed for the detection of virulent NDV including genotype VII (14–16). The above detection techniques require expensive instruments (e.g., Microplate readers, fluorescence detectors) and involve complex operations that necessitate professionally trained personnel. This limits the application potential of these methods for rapid, cost-effective on-site epizootic monitoring, particularly in primary veterinary laboratory with limited resources and inadequate skilled staff. Recently, the clustered regularly interspaced short palindromic repeats (CRISPRs) and associated nucleases constitute a serial of CRISPR/Cas systems. These systems have been widely applied in molecular diagnostics (17). The Cas nucleases most frequently utilized in CRISPR-based detection are Cas12 (also known as Cpf1), Cas13 (C2c2), and Cas14 (18, 19). Among them, the CRISPR/Cas13a (formerly C2c2) has the ability to recognize and cleave single-stranded RNA (ssRNA) targets without requiring a protospacer adjacent motif (PAM) sequence, which enables robust signal amplification for sensitive detection (20, 21). Despite their widespread application, the analytical sensitivity of these CRISPR/Cas systems falls below the thresholds required for clinical diagnostics, necessitating the incorporation of pre-amplification methods like (recombinase polymerase amplification) RPA or reverse-transcription loop-mediated isothermal amplification (RT-LAMP) to augment their sensitivity (22, 23). This integrated approach has been successfully applied for the rapid detection of multiple pathogens including Zika virus, Dengue virus, and SARS-CoV-2 (21, 24). CRISPR-Cas13a systems, when combined with isothermal amplification techniques such as RPA or RAA, have been widely applied for rapid and sensitive detection of animal viruses, including African swine fever virus (ASFV) (25), H9N2 avian influenza virus (AIV) (26), transmissible gastroenteritis virus (TGEV) (27). These assays typically achieve detection limits at the single-copy level within 30–60 min and offer field-deployable, equipment-free readouts, making them promising tools for point-of-care diagnostics in veterinary settings. Furthermore, the integration of CRISPR/Cas13a-based signal amplification with lateral flow dipstick (LFD)-mediated visual readout has been shown to further simplify the diagnostic workflow, potentially field-adaptable nucleic acid detection suitable for point-of-care settings (28). In this study, we established a rapid and sensitive detection method for genotype VII NDV by integrating RT-RPA, CRISPR/Cas13a, and LFD technologies. Using this assay, we successfully detected genotype VII NDV RNA. Consistency validation was performed using RT-qPCR as the reference method, and the results confirmed the reliability of this assay with clinical samples. Thus, this assay represents a simple and accurate tool for viral RNA detection in resource-limited settings.

## 2 Materials and methods

### 2.1 Viruses and clinical samples

The viruses of NDV strain of different genotype presented in Table 1, AIV(A/Chicken/Fujian/1.17 FZHX0102-C/2017), infectious bronchitis virus (IBV, strain Beaudette), avian leukosis virus type J (ALV-J, strain HN-1), Marek’s disease virus (MDV, strains CVI988), chicken infectious anemia virus (CIAV, strain JS2020-PFV), and fowl adenovirus serotype 4 (FADV-4, strain FJ20) were preserved by the Institute of Animal Science and Veterinary Medicine, Fujian Academy of Agricultural Sciences (Fuzhou, China). For clinical validation, 154 clinical samples were collected from live birds market from a routine surveillance program in Fujian province during September 2025 to May 2026, stored at −80 °C until use.

**Table 1:** Specificity of RT-RPA-CRISPR/Cas13a against different genotypes of field NDV isolates.

Isolate name	Genotype	Host	Collection date	GenBank ID	Virulence	RT-RPA-CRISPR/Cas13a
FP1/02	VII	Muscovy duck	2002	FJ872531	Virulent	+
PX2/03	VII	Muscovy duck	2003	JN653340	Virulent	+
DU-FJCL117	VII	duck	2011	KX765177	Virulent	+
DU-JXNC35	VII	duck	2012	KX765178	Virulent	+
DU-FJ-241	VII	duck	2014	KX765179	Virulent	+
LC213-1	I	duck	2010	KX765174	Low	−
LH11740	I	duck	2013	KX765175	Low	−
MH331-7	I	duck	2011	KX765176	Low	−
SP13	II	swine	2004	AY861659	Low	−
FM01/03	II	Mullar duck	2003	DQ023198	Low	−
HN311-43	II	duck	2014	KX765173	Low	−
XBT14	IX	Muscovy duck	2002	JX677561	Virulent	−
FJ1729	VI	Pigeon	2017	PZ339898	Virulent	−
FJ1745	VI	Pigeon	2017	PZ339899	Virulent	−
FJ1882	VI	Pigeon	2018	PZ339900	Virulent	−
FJ1997	VI	Pigeon	2019	PZ339901	Virulent	−

### 2.2 DNA/RNA extraction from viruses and clinical samples

The mix tissues samples collected from chicken or duck were homogenized in phosphate-buffered saline (PBS), subjected to freeze–thaw for three cycles, and centrifuged at 12,000 × g for 5 min to obtain the supernatant. Swab samples were vortexed to transfer the biological material to PBS, and briefly centrifuged, and the supernatant was collected. Viral DNA/RNA was extracted from viral stock of seven virus and 154 clinical samples supernatant using an EasyPure® Viral DNA/RNA Kit according to the manufacturer’s instructions (TransGen Biotech Co., Ltd., Beijing, China). The extracted DNA/RNA was stored at −80 °C until further use.

### 2.3 RT-RPA reactions

RT-RPA kit and LFD was obtained from EZassay Ltd. (Shenzhen, China) according to the manufacturer’s instructions. In brief, 20 μL of the reaction mixture contained 10 μL of rehydration buffer, 0.5 μL of each primer (20 μmol/L), 3 μL of RNA (template), 6 μL of RNase-free ddH_2_O, and 2 μL of the activator (magnesium acetate), RNase-free ddH_2_O was used as negative controls. Before running, the premix should be gently mixed by pipetting with filter tips. RPA tubes should be opened inside a biosafety cabinet located in a separate clean area to minimize exposure to airborne contaminants. Two μL of the activator is added. Subsequently, the reaction tube was incubated in a metal bath at 39 °C - 40 °C incubator for 20 min. After purifying the amplification products, the optimal RT-RPA primers were screened using 1% agarose gel electrophoresis.

### 2.4 Cas13a detection reactions

CRISPR/Cas13a reactions (total 20 μL) include 2 μL of cleavage buffer and 4 μL of Trans Mix, 0.5 μL of T7 RNA Polymerase, 1 μL of Cas13a protein (EZassay, Shenzhen, China), 2 μL of crRNA (1 μmol/L), 0.3 μL of RNA reporter-A (10 μmol/L), 5 μL of RT-RPA products, and RNase-free ddH_2_O to make a total volume of 20 μL. The reaction mixture was then placed in a fluorescence quantitative PCR instrument (BIOER, Hangzhou, China) and incubated at 37 °C for 15–30 min with the thermal lid set at 45 °C. Following the assay, the reaction tube was placed under a blue light source, and the fluorescence intensity was visually observed to screen for the optimal crRNA.

### 2.5 Lateral flow detection

For lateral flow detection, RNA reporter-B was used to replace RNA reporter-A in the CRISPR-Cas13a reaction system. The reaction product was diluted to a final volume of 100 μL and applied to the LFD test strip (EZassay, Shenzhen, China). On the test strip, the simultaneous appearance of the red detection line (T) and control line (C) indicates a positive result, while the presence of the red C line alone indicates a negative result. If the red C line is absent, the result is deemed invalid and retesting is required.

### 2.6 Construction of an RT-RPA-CRISPR/Cas13a system for NDV detection

**Figure 1:**
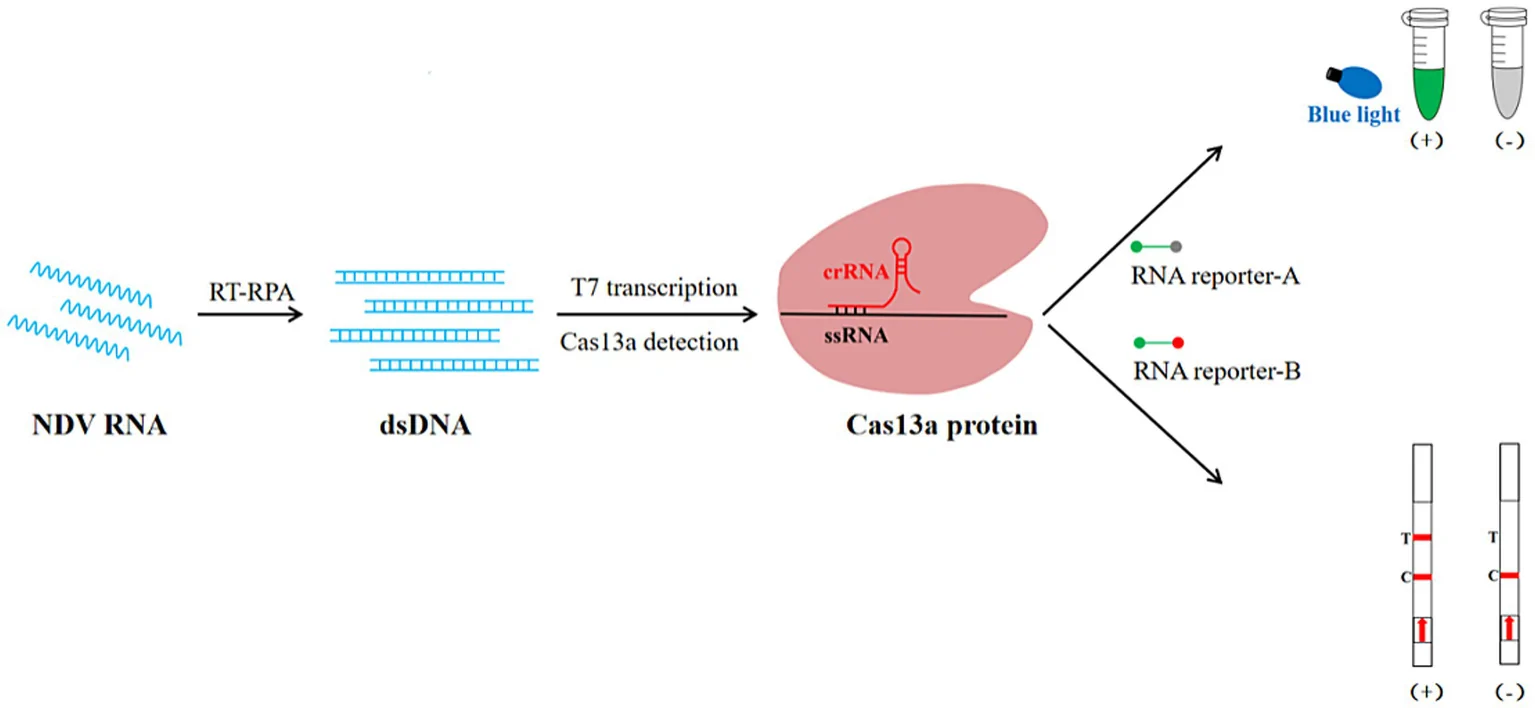
Schematic diagram of the RT-RPA-CRISPR/Cas13a-LFD workflow for NDV detection.

In this assay, target sequences were first amplified via RPA isothermal amplification. Subsequently, the amplicons harboring the T7 promoter were transcribed into target RNA *in vitro*. The pre-assembled Cas13a7 promoter were transcribed into target RNA *in vitro*. The pre-assembled cactivates the collateral cleavage activity of Cas13a. Consequently, the fluorescent reporter RNAs are degraded, generating detectable fluorescent signals. Upon completion of the reaction, the CRISPR/Cas13a assay with RNA reporter-A was read out for result interpretation by detecting the fluorescence signal under blue light irradiation, whereas the reaction mixture of the CRISPR/Cas13a-LFD assay with RNA reporter-B was introduced to the sample pad of the LFD, with results interpreted based on the control (C) line and test (T) line, the principle of which is illustrated in Figure 1. During the CRISPR/Cas13a reaction, the Cas13a-crRNA complex specifically recognized and cleaved the target dsDNA amplified by RT-RPA, which activated the trans-cleavage activity of Cas13a and thus triggered the non-specific degradation of the ssDNA probe. When RNA reporter-A was used as the reporter probe, the presence of the target induced probe cleavage, restoring the fluorescence signal of the quenched RNA reporter-A probe for visual result readout. In addition, when RNA reporter-B modified with FAM and biotin served as the reporter, it bound to the pre-immobilized FAM antibody-colloidal gold (red) and streptavidin-colloidal gold (red) on the LFD conjugate pad, forming an ssDNA complex (5′-gold-Ab-FAM-RNA reporter-Biotin-gold-3′). For negative samples, the anti-FAM antibody (secondary antibody) immobilized on the control (C) line preferentially captured the uncleaved RNA reporter-B complex, resulting in a single red signal on the C line only. For positive samples, the cleavage of RNA reporter-B reduced the chromogenic intensity of the C line, while simultaneously releasing the gold-streptavidin complex which then accumulated on the test (T) line, leading to the development of red signals on both the C and T lines. The LFD readout was completed within 5 mins, enabling visual result interpretation for on-site applications.

### 2.7 Design of primers, crRNAs, and ssRNA reporters

To enable specific detection of genotype VII NDV, we employed a two-step strategy that integrates RT-RPA amplification with Cas13a collateral cleavage activity. To design and screen RT-RPA primers and crRNA specific for genotype VII NDV, we performed multiple sequence alignments of the target regions against F gene sequences of diverse genotype VII sublineages (VII.1.1, VII.1.2, and VII.2) and other NDV genotypes (I, II, III, VI, IX, and XXI) retrieved from GenBank, based on sequence variations that discriminate genotype VII from other genotypes. Two types of single-stranded RNA reporter were synthesized: a fluorescence reporter (RNA reporter-A) for CRISPR-Cas13a-based fluorescence detection and an LFD reporter (RNA reporter-B) for LFD-based visualization. All primers, crRNA, RNA reporter (Table 2), and NDV F gene plasmids were synthesized by Sangon Biotech (Shanghai, China).

**Table 2:** The sequences of primers, crRNA and RNA reporter used in this study.

Name	Sequence (5′-3′)
NDV-RPA-F-F1	**TAATACGACTCACTATAG**CCATTAGAGGCATATAACAGAACACTGACTA
NDV-RPA-F-R1	CTGCCAATAACAGCACCTATAAAGCGT
NDV-RPA-F-R2	GCCGCTGTTGCAACCCCAAGAGCTACACTGC
NDV-RPA-F-R3	TGCTGCATCTTCCCAACTGCCACTGATAGTT
NDV-RPA-F-F2	**TAATACGACTCACTATAG**ATCCGCAAGATTCAAGGGTCTGTGTCCACG
NDV-RPA-F-F3	**TAATACGACTCACTATAG**CTGTGTCCACGTCTGGAGGAAGGAGACAA
NDV-RPA-F-F4	**TAATACGACTCACTATAG**TTATAGGTGCTGTTATTGGCAGTGTAGCTC
crRNA-1	GAUUUAGACUACCCCAAAAACGAAGGGGACUAAAACAAGGAGACAAAAACGCUUUAUAGGUG
crRNA-2	GAUUUAGACUACCCCAAAAACGAAGGGGACUAAAACCGUCUAGAGGAAGGAGACAAAAACGC
RNA reporter-A	FAM-UUUUUU-BHQ1
RNA reporter-B	FAM-AAUUUUUUAAUU-Biotin

The bold characters at the 5 end of primers NDV-RAP-F-F1/-F2/-F3/-F4 is the T7 promoter sequence.

### 2.8 Optimization of CRISPR/Cas13a detection reactions

The CRISPR/Cas13a reactions were conducted with different concentrations of Cas13a (25, 50, 100, 150, and 200 nmol/L) and crRNA (25, 50, and 100 nmol/L). The fluorescence signal of each reaction product was detected to identify the optimal concentrations of the two components.

### 2.9 S**ensitivity of RT-RPA-CRISPR/Cas13a detection reactions**

For sensitivity testing, the F gene plasmid of genotype VII NDV was diluted to different concentrations ranging from 6 × 10^7^ to 6 copies/μL, and 3copies/μL. And assays results were observed through both fluorescence signal detection and LFD methods, with ddH_2_O serving as the negative control. After the reaction was completed, the fluorescence intensity of each reaction mixture was observed under a blue light illuminator with a wavelength of 450 nm, or validated via LFD assay.

### 2.10 Specificity of RT-RPA-CRISPR/Cas13a detection method

For analytical specificity assessment of the RT-RPA-CRISPR/Cas13a assay, nucleic acids were extracted from a panel of poultry pathogens, including NDV, AIV, infectious bronchitis virus (IBV), avian leukosis virus (ALV), Marek’s disease virus (MDV), chicken infectious anemia virus (CIAV), and fowl adenovirus serotype 4 (FAdV-4). Fifteen NDV clinical isolates of different genotypes (Table 1) reserved in our laboratory were used to further confirm the genotype specificity of the assay. As described above, the specificity of the RT-RPA-CRISPR/Cas13a assay was evaluated using a blue light illuminator and LFD.

### 2.11 Validation of RT-RPA-CRISPR/Cas13a assay for sample detection

To assess the clinical utility of the newly developed RT-RPA-CRISPR/Cas13a assay, we performed parallel testing of 154 clinical samples (60 chicken swabs, 60 duck swabs, 19 chicken tissue samples, and 15 duck tissue samples) using our established assay and a reference RT-PCR method targeting the NDV F gene. The primer pairs used for F gene RT-PCR amplification were as follows: F: 5′-ACACGGGTAGAAGATTCTGGATCCCGGTTG-3′, R:5′-CACAGCCTCATTGGTTGCAGCAATGC-3′. All RT-PCR amplification products from positive samples were subsequently sequenced, and viral genotype assignments were confirmed by phylogenetic analysis based on F gene sequences of different NDV genotypes. The detection results of the newly developed assay were compared with the RT-PCR-based genotyping results to evaluate the diagnostic effectiveness and specificity of our method.

### 2.12 Statistical analysis

GraphPad Prism 10.1.2 software (GraphPad Software, Inc.) was utilized to analyze the data. Statistical significance was evaluated through the application of two-tailed t-tests. The results were shown as mean ± standard error of the mean (SEM) based on three independent experiments, with *p* values lower than 0.05 being considered significant.

The 95% confidence intervals for diagnostic indices including sensitivity, specificity, positive predictive value (PPV) and negative predictive value (NPV) were calculated using the Wilson score method. Cohen’s Kappa statistic was applied to evaluate the agreement between the RT-RPA-CRISPR/Cas13a-(LFD) assay and sequencing results.

## 3 Results

### 3.1 S**creening of the RT-RPA primers and crRNA**

**Figure 2:**
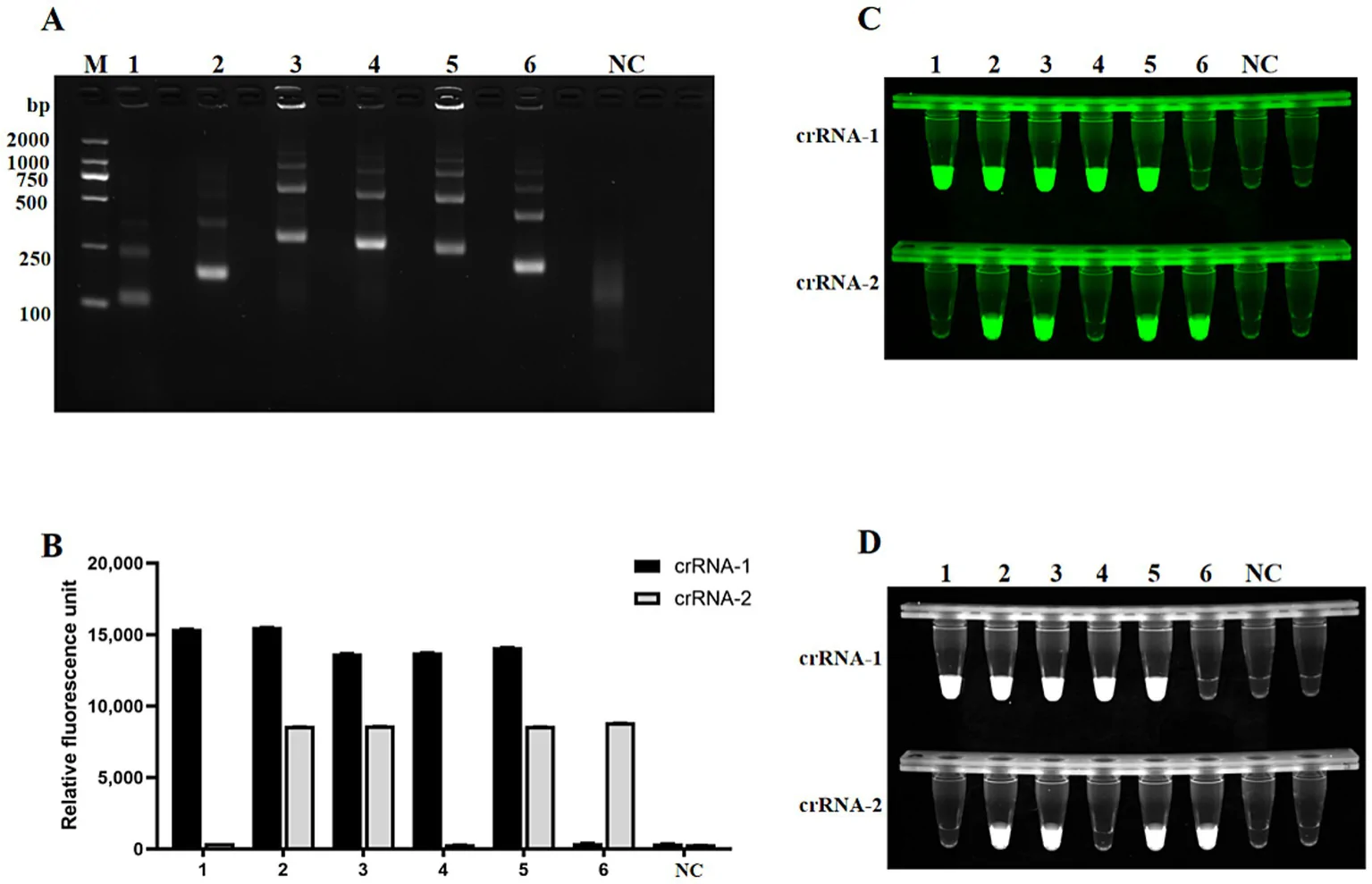
Screening of primers and crRNA for the RT-RPA-CRISPR/Cas13a system. **(A)** RT-RPA products verified by 1% agarose gel electrophoresis. M: DL2000 DNA marker; 1: F1R1; 2: F1R2; 3: F1R3; 4: F2R4; 5: F3R4; 6: F4R4. Each reaction included a negative control with RNase-ddH_2_O as the template. CRISPR/Cas13a fluorescence detection was performed using six primer sets. Fluorescence values **(B)**, histogram of fluorescence values **(C)**, and detection using a UV transilluminator allowed examination with the naked eye **(D)** were shown for CRISPR/Cas13a detection using six primer sets.

Based on multiple sequence alignment of primer binding sites and crRNA target regions from different genotype NDV, seven primers (yielding six pairwise combinations) and two CRISPR RNA (crRNA) were designed. The RT-RPA primers and crRNAs combinations were selected based on its amplification, clarity of product on agarose gel electrophoresis and RT-RPA-CRISPR/Cas13a fluorescence assays. Among these, the combination of RT-RPA primers (NDV-RPA-F-F1 and NDV-RPA-F-R2) and the crRNA-1 demonstrated the most robust amplication performance (Figure 2).

### 3.2 Optimization of Cas13a and crRNA concentrations

**Figure 3:**
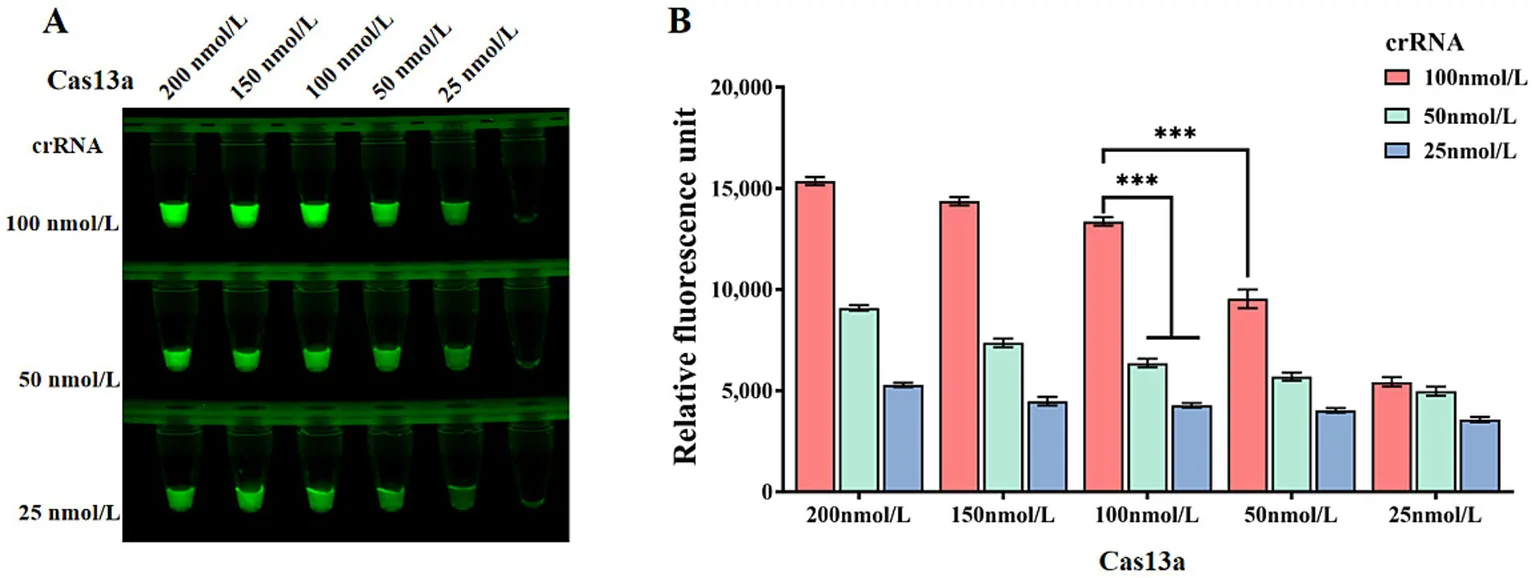
Optimization of the RPA and CRISPR/Cas13a system. **(A)** Fluorescence intensity based on CRISPR/Cas13a reaction mediated by different concentrations of Cas13a and crRNA. **(B)** Histogram of fluorescence values.

To optimize the concentrations of Cas13a and crRNA for the CRISPR/Cas13a system, we tested a gradient of Cas13a concentrations (25, 50, 100, 200 nmol/L) and crRNA concentrations (25, 50, 100 nmol/L). As shown in Figures 3A,B, the fluorescence intensity of the 100 nmol/L Cas13a group was significantly higher than that of the 50 nmol/L group (*p* < 0.001). Under these conditions, the 100 nmol/L crRNA group also exhibited a markedly higher fluorescence intensity than the 50 nmol/L and 25 nmol/L crRNA groups (*p* < 0.001). Based on these results, both Cas13a and crRNA were used at a concentration of 100 nmol/L in all subsequent experiments.

### 3.3 Sensitivity of RT-RPA-CRISPR/Cas13a method

**Figure 4:**
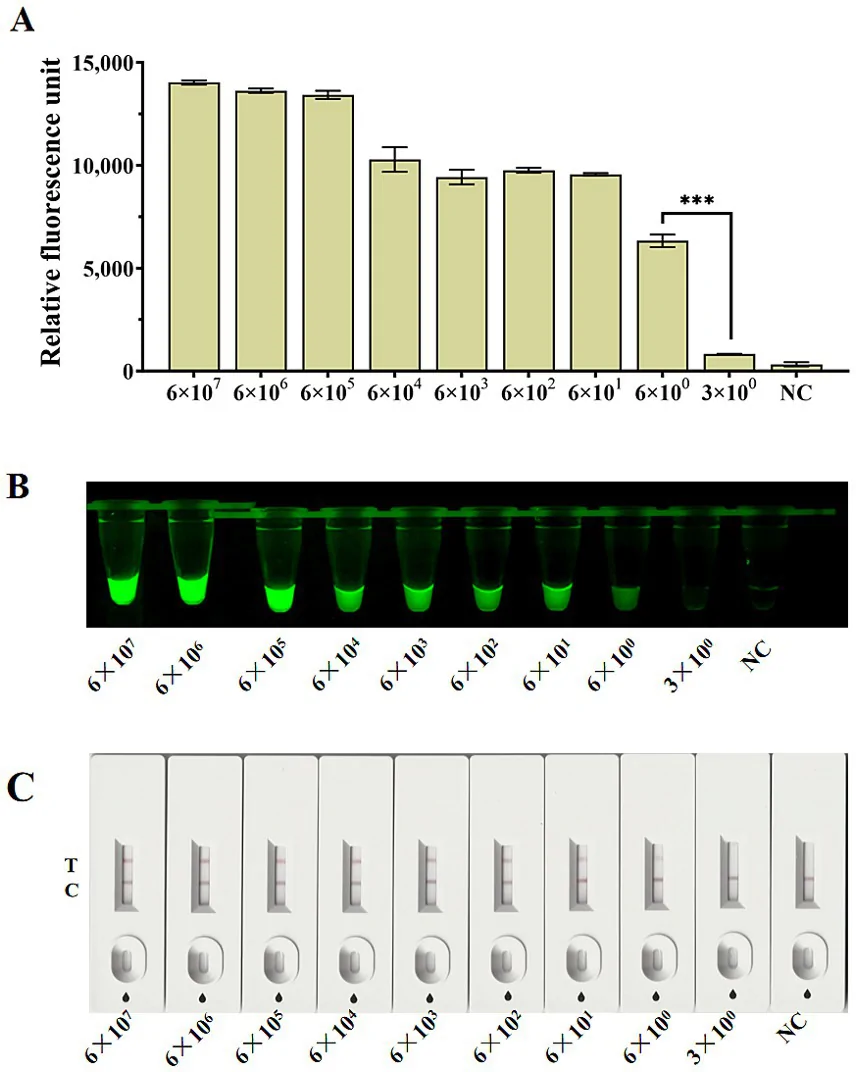
Sensitivity assessment of the RT-RPA-CRISPR/Cas13a system coupled with LFD. **(A)** Histogram of NDV detection limits by RT-RPA-CRISPR/Cas13a. **(B)** Detection using a blue light transilluminator. **(C)** Analytical sensitivity of RT-RPA-CRISPR/Cas13a-LFD in NDV detection.

The analytical sensitivity of the RT-RPA-CRISPR/Cas13a assay was assessed using serially diluted NDV F gene plasmids at concentrations of 6 × 10^7^ to 6 and 3 copies/μL, alongside RNase-free ddH_2_O as the negative control. Following RPA amplification, the CRISPR/Cas13a-mediated fluorescent assay exhibited a consistent limit of detection (LOD) of 6 copies/μL template across three independent replicate experiments (Figures 4A,B). Concordantly, parallel LFD assays exhibited a distinct, visible T line signal at the concentration of 6 copies/μL (Figure 4C).

### 3.4 Specificity of RT-RPA-CRISPR/Cas13a detection method

**Figure 5:**
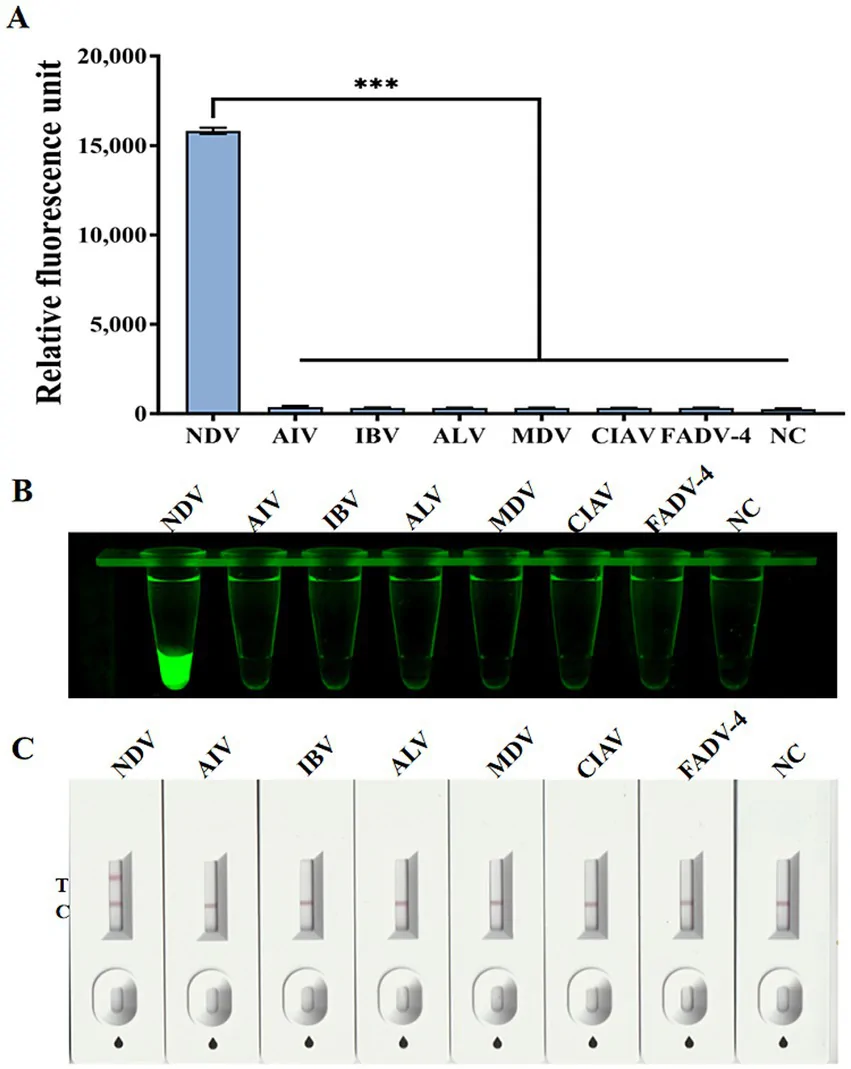
Specificity assessment of the RT-RPA-CRISPR/Cas13a assay. **(A)** Histogram of NDV detection specificity by RT-RPA-CRISPR/Cas13a. **(B)** Detection using a blue light transilluminator. **(C)** Analytical sensitivity of RT-RPA-CRISPR/Cas13a-LFD in NDV detection.

The specificity of the established RT-RPA-CRISPR/Cas13a assay was evaluated using six prevalent avian pathogens, including AIV, IBV, ALV, MDV, CIAV, and FAdV-4. No cross-reactivity was detected by either fluorescence observation or lateral flow dipstick (LFD) visualization (Figure 5), demonstrating the high specificity of this assay toward NDV.

Further specificity verification was performed with NDV strains belonging to genotypes I, II, VI, VII, and IX. Positive signals were exclusively obtained for genotype VII, whereas no cross-reactivity occurred with the other genotypes (Table 1). Collectively, these results confirm that the developed assay possesses high specificity for detection of genotype VIINDV.

### 3.5 Validation of the assay for clinical sample detection

To evaluate detection performance, 154 clinical samples were screened using a universal NDV RT-PCR assay and the newly developed RT-RPA-CRISPR/Cas13a assay. The RT-PCR assay identified 25 NDV-positive samples, comprising four chicken swabs, seven duck swabs, six chicken tissue samples, and eight duck tissue samples. In contrast, the RT-RPA-CRISPR/Cas13a assay specifically detected 10 genotype VII NDV-positive samples, including one chicken swab, two duck swabs, four chicken tissue samples, and three duck tissue samples. Subsequent sequencing and phylogenetic analysis of universal NDV RT-PCR products verified that all positive samples identified by the RT-RPA-CRISPR/Cas13a assay were accurately classified as NDV genotype VII (Table 3).

**Table 3:** Results of clinical sample test.

Sample type	Sample number	Positive number		Number of genotype VII^c^
		RT-RPA-CRISPR/Cas13a	RT-PCR	
Chicken Swab^a^	60	1	4	1
Duck Swab	60	2	7	2
Chicken tissue^b^	19	4	6	4
Duck tissue	15	3	8	3

a Oral/Cloacal Swab.

b tissue was mixed sample contain liver, spleen, kidney and lung.

c Genotype identification was performed by sequencing the NDV F gene fragment of all positive samples obtained from RT-PCR and conducting phylogenetic analysis.

Using sequencing-based phylogeny as the reference standard, we constructed a 2 × 2 contingency table (Table 4) to assess the diagnostic performance of the novel assay. Parallel testing of the 154 field samples yielded complete concordance between the novel assay and the reference method, with 10 true positives and 144 true negatives. Statistical evaluation (Table 5) showed a diagnostic sensitivity of 100.00% (95% CI: 72.25–100.00%) and specificity of 100.00% (95% CI: 97.38–100.00%), with both positive and negative predictive values of 100.00% (95% CI: 72.25–100.00% and 97.38–100.00%, respectively). The Cohen’s kappa coefficient was 1.00, indicating perfect diagnostic agreement between the novel assay and the reference method.

**Table 4:** RT-RPA-CRISPR/Cas13a assay with sequencing results.

Assay results	Sequencing results		Total
	Positive	Negative	
Positive	10	0	10
Negative	0	144	144
Total	10	144	154

**Table 5:** Diagnostic performance of the RT-RPA-CRISPR/Cas13a assay.

Parameter	Value	95% confidence interval
Sensitivity (%)	100.00	72.25–100.00
Specificity (%)	100.00	97.38–100.00
Positive predictive value (%)	100.00	72.25–100.00
Negative predictive value (%)	100.00	97.38–100.00
Cohen’s Kappa	1.00	—

## 4 Discussion

NDV exhibits high genetic diversity, and more than 20 genotypes have been identified to date (16). Class II genotypes of NDV are responsible for most global ND outbreaks and have caused at least five panzootics worldwide. Although genotype V, VI, VIII and XIII also pose considerable threats to the poultry industry, virulent genotype VII NDV strains predominated and are most frequently isolated from many outbreaks among chickens (15, 29, 30). Genotype VII NDV were responsible for the fourth and fifth panzootics (7, 31, 32), specifically sub-genotypes Genotype VII.1.1 and VII.2 (10, 33, 34). The genotype VII NDV is originated in Southeast Asia, with the earliest known outbreaks beginning around 1985 and is believed to be the main contributors to most ND outbreaks across Eurasia, Africa, South America (10). A rapid and accurate diagnosis is critical for effective epidemic prevention and control. In this study, we developed a rapid on-site detection assay for genotype VII virulent NDV by combining RPA and CRISPR/Cas13a technology. This method enables rapid nucleic acid amplification under isothermal conditions (39 °C) via RPA, and leverages the RNA-guided collateral cleavage activity of Cas13a to complete detection within 40 min, with a limit of detection of 6 copies/μL. The results can be visualized by fluorescence signals or LFD band patterns.

**Figure 6:**
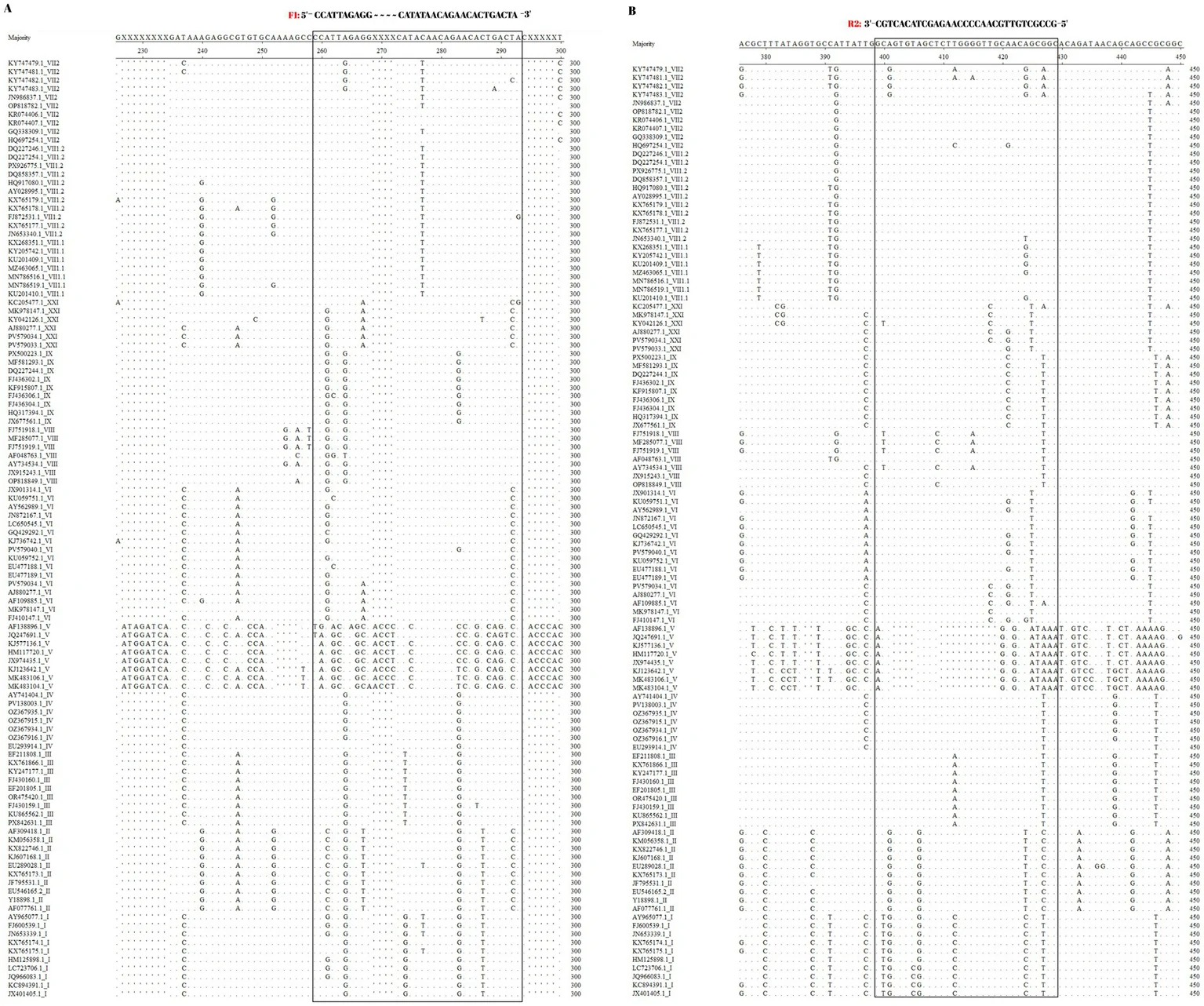
In silico alignment of RPA primer with different genotype NDV. **(A)** Alignment result of RPA primer NDV-RPA-F-F1 with target regions of F gene across different genotype NDV; **(B)** Alignment result of RPA primer NDV-RPA-F-R2 with target regions of F gene across different genotype NDV.

**Figure 7:**
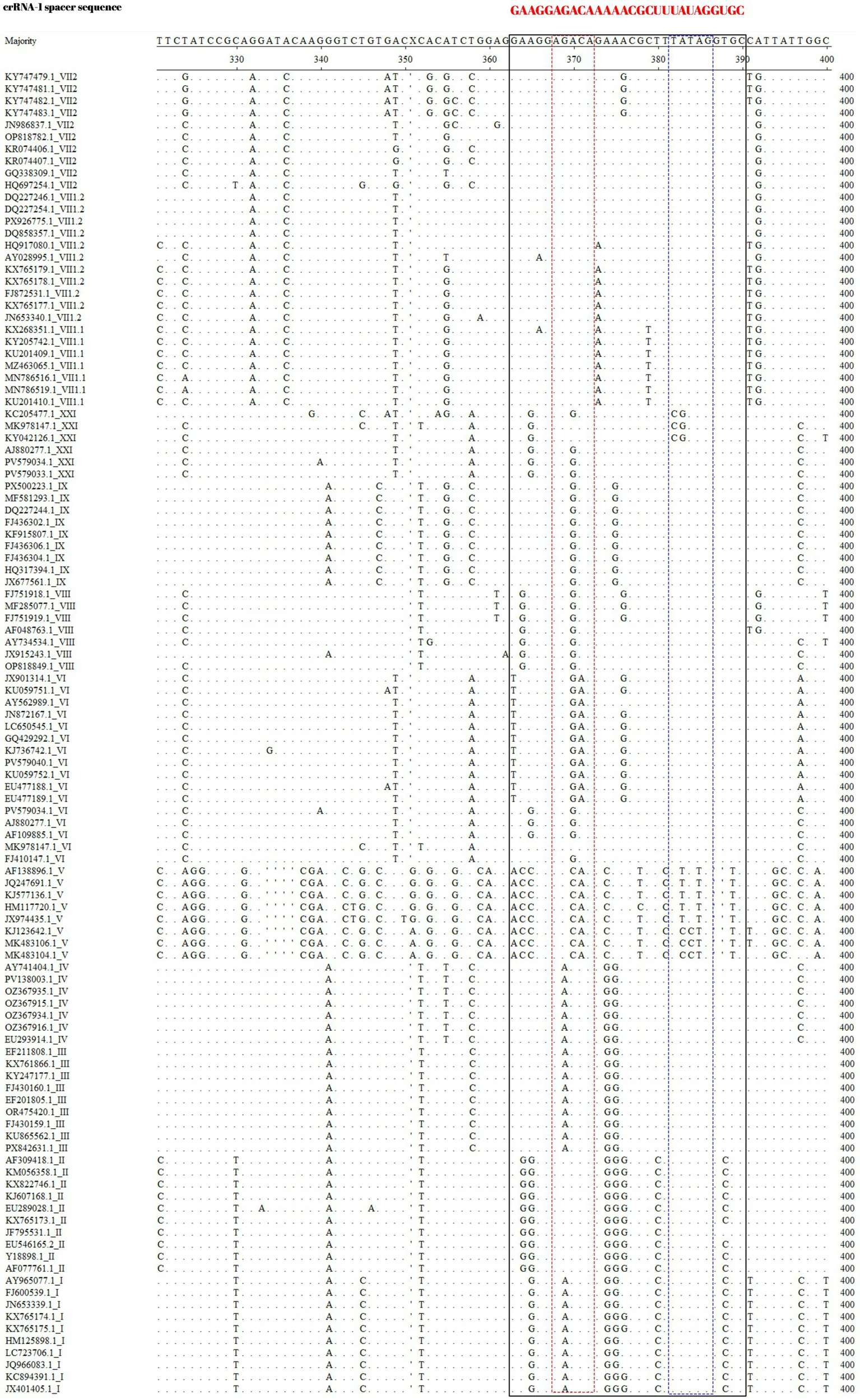
In silico alignment of crRNA with different genotype NDV. The red dashed box and the blue dashed box indicates the core sensitive motifs located at positions 6–10 nt and 20–24 nt within the spacer sequence of crRNA-1,respectively.

In this study, the RT-RPA primers and crRNA were designed and screened according to sequence variations distinguishing genotype VII NDV from other NDV genotypes. The primers (NDV-RPA-F-F1 and NDV-RPA-F-R2; Figure 6) and crRNA (crRNA-1; Figure 7) target regions are highly conserved among genotype VII strains, while showing sufficient mismatches with other genotypes NDVstrains to ensure specificity. Especially, the core sensitive motifs located at positions 6–10 nt and 20–24 nt within the spacer sequence of crRNA-1 (Figure 7) are perfectly complementary to the target sequences of genotype VII NDV (subgenotypes VII 1.1, VII 1.2 and VII 2), and no consecutive dinucleotide mutations were observed across the full binding region. By contrast, virulent strains belonging to genotypes VI, IX and XXI carry base mutations within the two core sensitive regions. Such mismatches hinder the activation of the trans-cleavage activity of Cas13a even after crRNA-1 hybridizes with the amplified target, thereby enabling the crRNA-1-guided CRISPR-Cas13a system to specifically discriminate genotype VII from other virulent NDV genotypes. Nevertheless, spontaneous base variations occur at non-core sensitive sites: positions 4 nt and 17 nt in VII 1.1, positions 4 nt and 11 nt in VII 1.2, as well as positions 11 nt and 14 nt in VII 2. Mutations in these non-core regions cannot completely abolish the detection reaction, yet they reduce the overall reaction efficiency and compromise the limit of detection of this assay. This defect could be further optimized by introducing degenerate bases at these variable sites in subsequent crRNA redesign (35).

Notably, the study only was validated on 10 positive samples with consistency against RT-PCR. The limited number of clinical genotype VII NDV-positive samples (*n* = 10) resulted in a relatively wide confidence interval for the calculated sensitivity. The relatively low detection rate observed in this study could be explained by the sample sources and the types of specimens collected. All 154 samples were obtained from routine surveillance at live bird markets. Among them, 77.9% (120/154) samples were swabs from apparently healthy birds, only 22.1% (34/154) were tissue samples from suspected cases. In China, the strategy of intensive vaccination combined with culling of infected birds has effectively curbed clinical outbreaks caused by genotype VII NDV (36). This widespread vaccination has severely limited the circulation of genotype VII NDV in field. The limited number of positives genuinely reflects the low prevalence of genotype VII NDV in the surveyed population, not insufficient assay sensitivity. Future studies will incorporate a larger cohort of field samples to further validate and consolidate the practical detection performance of this assay in clinical applications.

By refining the preprocessing protocols and amplification systems employed in this study, the overall workflow can be further streamlined, reducing dependence on specialized equipment and controlled laboratory settings. These improvements will enhance the convenience, rapidity, and field-deployability of the detection assay, advancing its development toward portable, real-time, and point-of-care diagnostic capabilities for grassroots applications (37–39). Accordingly, this work will provide more practical technical support for the rapid screening and precise prevention and control of viral infections in resource-limited field settings. The approach established in this study cannot cover all the currently circulating virulent genotypes, and is specifically targeted at the genotype VII only. Future work should focus on developing multiplex detection assays that encompass most of the virulent NDV genotypes to adapt to the dynamic epidemiological evolution of NDV.

## Data Availability

The raw data supporting the conclusions of this article will be made available by the authors, without undue reservation. The datasets presented in this study can be found in online repositories. The names of the repository/repositories and accession number(s) can be found at: https://www.ncbi.nlm.nih.gov/genbank/, AY861659, DQ023198, FJ872531, JN653340, JX677561, KX765173-KX765179, and PZ339898-PZ339901.
